# TsrA modulates type III secretion system 2 expression as a co-regulator of H-NS in *Vibrio parahaemolyticus*

**DOI:** 10.1128/jb.00556-25

**Published:** 2026-05-28

**Authors:** Tan Paramita Wibowo Sutanto, Andre Pratama, Eiji Ishii, Tetsuya Iida, Shigeaki Matsuda

**Affiliations:** 1 Department of Bacterial Infections, Research Institute for Microbial Diseases, The University of Osaka 34822, Osaka, Japan; 2 Department of Infection Metagenomics, Bioinformatics Center, Research Institute for Microbial Diseases, The University of Osaka 320555, Osaka, Japan; 3 Center for Infectious Disease Education and Research, The University of Osaka 13013, Osaka, Japan; Southern University of Science and Technology, Shenzhen, Guangdong, China

**Keywords:** *Vibrio parahaemolyticus*, gene regulation, type III secretion system, H-NS, virulence regulation, gene silencing

## Abstract

*Vibrio parahaemolyticus*, a gram-negative marine bacterium, is a major cause of seafood-borne gastroenteritis worldwide. This pathogen relies on type III secretion system 2 (T3SS2), which is encoded on a pathogenicity island, for its enteropathogenicity. Expression of T3SS2 is activated by a regulatory pathway centered on the transcriptional activator VtrB, which is antagonized by the xenogeneic silencer, histone-like nucleoid-structuring protein (H-NS). However, the complete transcriptional network is not yet fully understood. In this study, we identified TsrA as a negative regulator of T3SS2 gene expression. TsrA is a small protein conserved among *Vibrio* species that lacks a putative DNA-binding motif but has been implicated in the regulation of virulence genes in *Vibrio cholerae*. In *V. parahaemolyticus*, deletion of *tsrA* increased VtrB production and T3SS2 secretion, thereby enhancing T3SS2-dependent pathogenicity. Transcription of *vtrB* occurs via a two-step activation process, in which TsrA affects the primary activation step, thereby modulating VtrB production. We further provide experimental evidence that TsrA physically interacts with H-NS via its C-terminal region, which correlates with its regulatory activity on *vtrB* expression. A systematic mutational analysis of the C-terminal 26 residues revealed several residues critical for TsrA regulatory activity. Moreover, the regulatory effect of TsrA on T3SS2 gene expression was dependent on H-NS, demonstrating that TsrA functions in concert with H-NS. Thus, our findings provide new insights into the regulatory mechanisms of virulence gene expression in *V. parahaemolyticus* by defining the role of TsrA in this network, while also placing TsrA among H-NS co-regulators.

**IMPORTANCE:**

Nucleoid-associated proteins (NAPs) play key roles in virulence gene regulation in bacteria. The best-studied NAP is H-NS, which often functions with co-regulators to fine-tune gene expression. TsrA, a small protein lacking a DNA-binding motif conserved among *Vibrio* species, has been suggested to be functionally related to H-NS in *Vibrio cholerae*, although its mechanism remains unknown. Here, we demonstrate that TsrA negatively regulates the expression of type III secretion system 2 (T3SS2), a major virulence determinant of *Vibrio parahaemolyticus*, an important seafood-borne pathogen. TsrA modulates the transcription of *vtrB*, which encodes the essential activator for T3SS2 expression, through direct physical interaction with H-NS. Our findings reveal a molecular link between TsrA and H-NS, providing mechanistic insights into NAP- and TsrA-mediated regulation of virulence in *Vibrio*.

## INTRODUCTION

*Vibrio parahaemolyticus* is a gram-negative marine bacterium widely known as a major causative agent of seafood-borne gastroenteritis (1). Over the past 25 years, infections caused by *V. parahaemolyticus* have spread globally, with increasing reports across diverse geographic regions. Since the incidence of *V. parahaemolyticus* infections correlates with elevated seawater temperatures, ongoing climate change is expected to further exacerbate the global risk of *V. parahaemolyticus* infections (2). The enteropathogenicity of this organism requires type III secretion system 2 (T3SS2). The type III secretion system (T3SS) is a specialized protein secretion system widely employed by gram-negative pathogens and symbionts (3–5). T3SS forms a needle-like structure that extends from the bacterial surface, with a translocon complex at its tip that inserts into the host cell membrane to deliver effector proteins directly into the host cytoplasm, where they manipulate host cell functions to promote bacterial pathogenesis and symbiosis (6). In *V. parahaemolyticus*, T3SS2 secretes 11 effectors identified hitherto and contributes to gastrointestinal pathogenesis (7–9).

T3SS2-related genes are clustered on an 80 kb pathogenicity island known as the *V. parahaemolyticus* pathogenicity island (Vp-PAI). The expression of genes within the Vp-PAI is governed by two membrane-bound transcriptional regulators: VtrA and VtrB (10, 11). VtrA forms a complex with its co-transcribed protein, VtrC (12). The VtrA/VtrC complex binds near the −35 element of the *vtrB* promoter to initiate *vtrB* transcription. This activation requires ToxR, which binds upstream of the VtrA-binding site in the *vtrB* promoter (5). Following this primary activation of *vtrB* expression, VtrB amplifies its own expression through a positive feedback loop, in which the transcription of the VtrB-regulated operon located upstream of *vtrB* extends across the terminator into the *vtrB* gene in a read-through manner (13). This autoregulatory loop results in the secondary activation of *vtrB* gene expression and further enhances T3SS2 gene expression, which is critical for the pathogenicity of *V. parahaemolyticus*. Conversely, *vtrB* gene expression is repressed by the global transcriptional repressor H-NS (5). H-NS binds upstream of the *vtrB* gene, where it antagonizes the binding of VtrA and ToxR to the *vtrB* promoter. Thus, the complex regulatory network coordinates the fine-tuned expression of T3SS2 genes in *V. parahaemolyticus*.

TsrA is a small protein (~94 amino acids) conserved among *Vibrio* species (14). In *Vibrio cholerae*, TsrA has been described as a negative regulator of virulence genes, including cholera toxin, toxin-coregulated pilus, and type VI secretion system genes (14–17). Previous reports have shown that in *V. cholerae*, TsrA-regulated genes are localized within horizontally acquired genetic islands, suggesting a role for TsrA in xenogeneic silencing against foreign genes, similar to that of H-NS (16). However, the role of TsrA in other pathogenic *Vibrio* species has not yet been explored. Given that H-NS is involved in the expression of T3SS2 genes encoded within a horizontally acquired pathogenicity island in *V. parahaemolyticus*, we hypothesized that TsrA similarly influences this virulence determinant. In this study, we examined the role of TsrA in regulating T3SS2 gene expression and *V. parahaemolyticus* pathogenicity.

## RESULTS

### TsrA negatively affects T3SS2 gene expression

**Fig 1:**
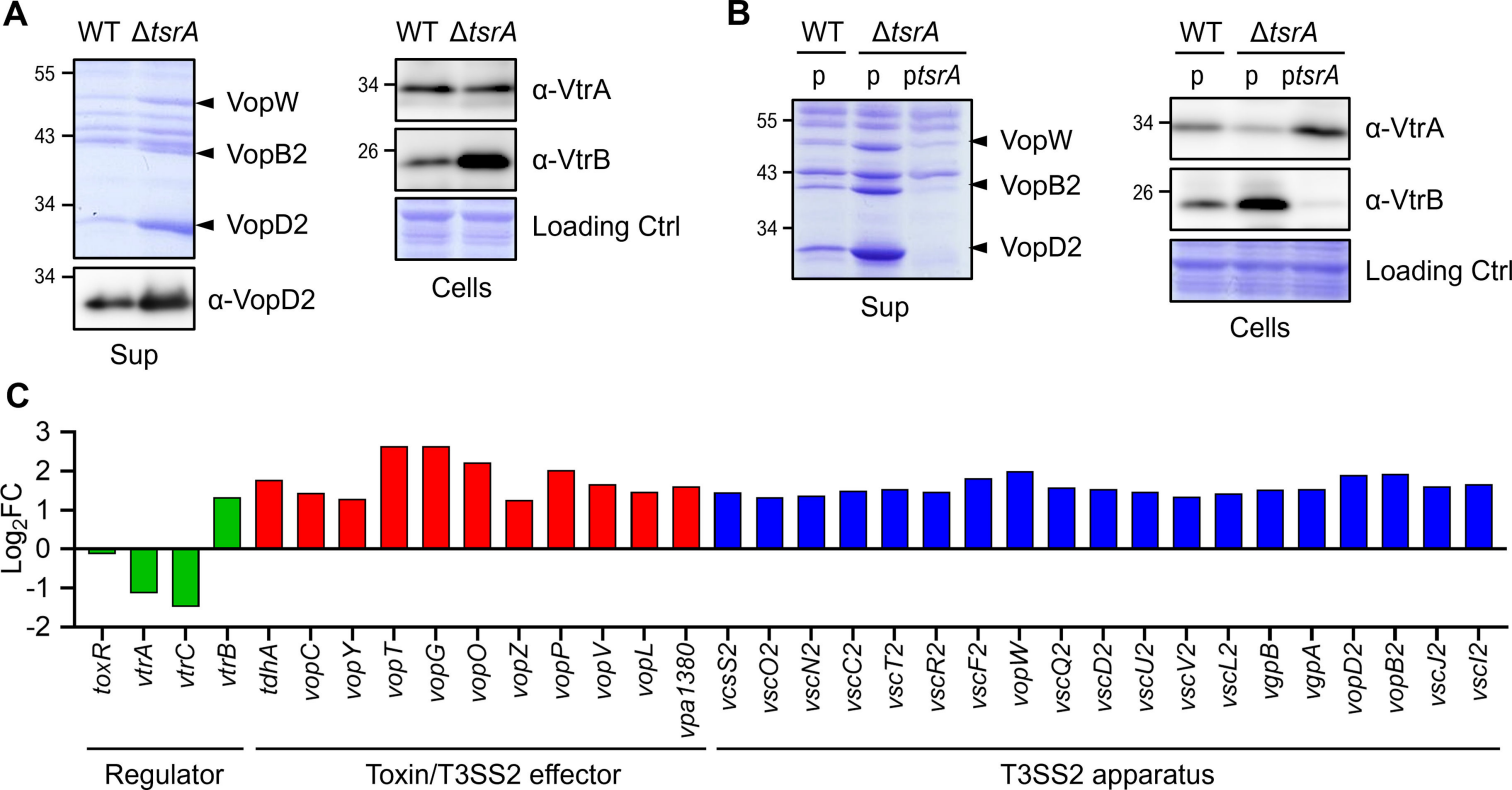
TsrA negatively affects the expression of T3SS2 genes. (**A**) The *tsrA*-deleted strain (Δ*tsrA*) showed increased levels of T3SS2-related proteins. *V. parahaemolyticus* WT and Δ*tsrA* strains were grown in Luria–Bertani (LB) medium at 37°C to an OD_600_ of 1.8. Secreted proteins in culture supernatants (Sup) were analyzed by SDS-PAGE followed by Coomassie Brilliant Blue (CBB) staining, with T3SS2-secreted proteins (VopW, VopB2, and VopD2) indicated by arrowheads, or by immunoblotting for VopD2. Bacterial cell lysates (Cells) were analyzed by immunoblotting for VtrA and VtrB, with proteins visualized by CBB staining serving as a loading control. (**B**) Complementation of *tsrA* gene restored the repressive effect on T3SS2-related proteins. *V. parahaemolyticus* WT and Δ*tsrA* strains harboring pSA19 empty vector (indicated as p), and Δ*tsrA* harboring pSA19-*tsrA* with the endogenous promoter (indicated as p*tsrA*) were grown in LB medium at 37°C to an OD_600_ of 1.8. Sup was separated by SDS-PAGE, followed by CBB staining. Cells were analyzed by immunoblotting for VtrA and VtrB. Cell lysate proteins visualized by CBB staining served as a loading control. (**C**) Differentially expressed genes related to T3SS2 in the Δ*tsrA* mutant compared to the WT strain. Total RNA was extracted from *V. parahaemolyticus* WT and Δ*tsrA* strains grown in LB medium at 37°C to an OD_600_ of 0.8–1.0 and analyzed using RNA-Seq.

To test this hypothesis, we first constructed a *tsrA* (*VP3018*)-deleted strain derived from the *V. parahaemolyticus* reference strain RIMD2210633 (Δ*tsrA*) and examined the secretion of T3SS2-related proteins into the culture supernatant to monitor the secretory activity of T3SS2 (Fig. 1A). In the wild-type (WT) strain, VopD2, VopB2, and VopW, components of the T3SS2 translocon complex, were clearly detected among the T3SS2-secreted proteins. Notably, the secretion of T3SS2-related proteins was further increased in Δ*tsrA* compared to that in WT, indicating that deletion of *tsrA* leads to the hypersecretion of T3SS2. Given the critical roles of VtrA and VtrB in T3SS2 gene expression, we also examined their expression by immunoblotting (Fig. 1A). The production of VtrB was increased in Δ*tsrA* compared to that in WT, whereas the protein level of VtrA was rather decreased in Δ*tsrA* compared to that in WT. Considering the hierarchical relationship between VtrA and VtrB, the elevated expression of VtrB likely accounts for the enhanced T3SS2 expression, despite the reduced level of VtrA. In addition, deletion of *vtrB* in the Δ*tsrA* mutant abolished the reduction in VtrA levels, suggesting that this decrease is mediated by VtrB activation (Fig. S1). Complementation of Δ*tsrA* with a plasmid-borne *tsrA* gene abolished the increased expression of VtrB and the hypersecretion of T3SS2 (Fig. 1B). Consistent with the protein production and secretion analyses, transcriptome profiling using RNA-seq showed that the expression of T3SS2-related genes was upregulated in the Δ*tsrA* strain compared to that in the WT strain (Fig. 1C). Among the known T3SS2 regulators, only *vtrB* showed an increased expression. These results indicate that TsrA negatively regulates T3SS2 gene expression by modulating *vtrB* expression.

### TsrA affects primary activation of *vtrB* gene expression

**Fig 2:**
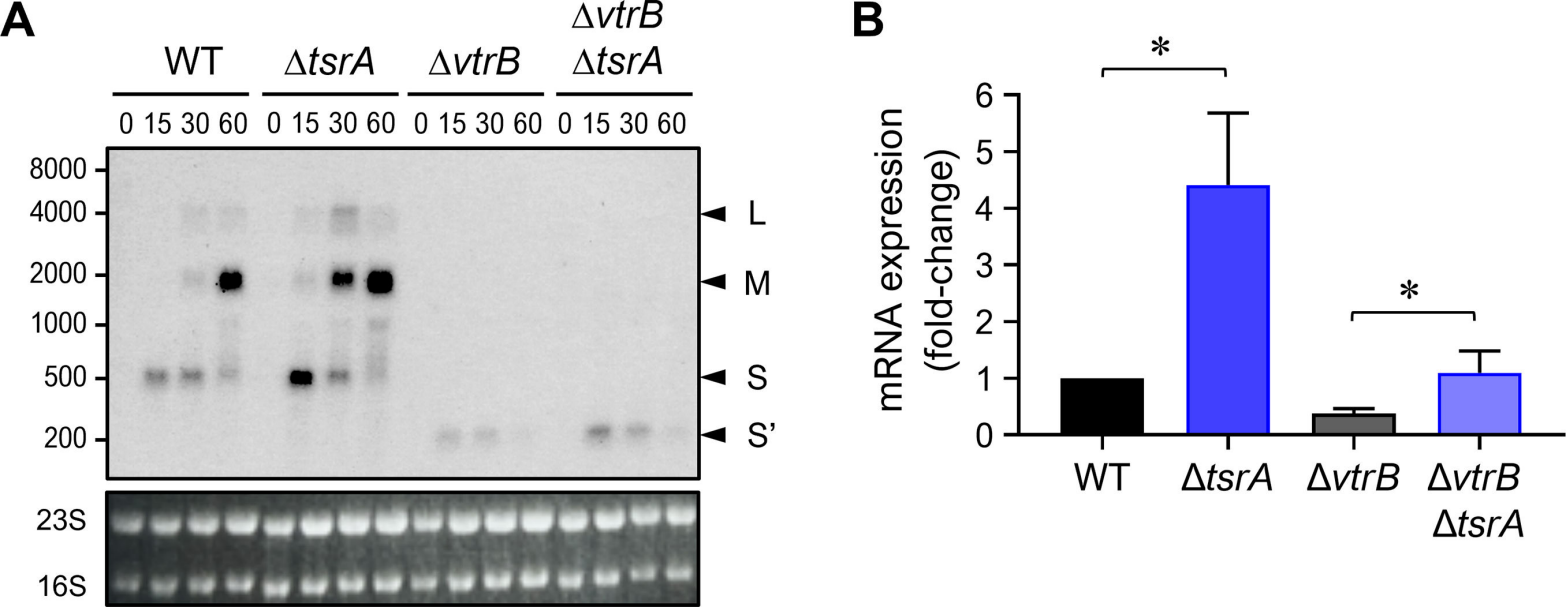
TsrA affects the initial activation of *vtrB* transcription. (**A**) Time-course analysis of *vtrB* transcripts in *V. parahaemolyticus* strains. The indicated strains were grown to an OD_600_ of 0.8 at 25°C, a non-permissive condition for *vtrB* expression, and then shifted to 37°C to induce *vtrB* gene expression. RNA was extracted at 0, 15, 30, and 60 min after induction, and northern blotting was performed using a *P_vtrB_* probe. L, 4,000 nt L transcript; M, 2,000 nt M transcript; S, 700 nt S transcript; S’, S transcript containing *vtrB* deletion. 23S rRNA and 16S rRNA were used as loading controls. (**B**) Relative expression of the *vtrB* promoter region in *V. parahaemolyticus* WT, Δ*tsrA*, Δ*vtrB*, and Δ*vtrB* Δ*tsrA* strains. Total RNA samples collected 15 min after induction in panel A were used for qRT-PCR analysis. Expression levels were normalized to *recA* and expressed relative to those of the WT strain. *, *P* < 0.01 by student’s *t*-test.

A previous study showed that *vtrB* gene expression is conferred by a two-step transcriptional activation, in which VtrA-mediated primary activation of *vtrB* transcription from the *vtrB* promoter is followed by secondary activation by VtrB-mediated read-through transcription from the operon upstream of *vtrB* (13). To dissect the mechanisms by which TsrA represses *vtrB* gene expression, we performed a time-course northern blot analysis of *vtrB* transcripts using a probe against the *vtrB* promoter (Fig. 2A). RNA was collected at multiple time points following a temperature shift from 25°C (non-permissive for *vtrB* expression) to 37°C (permissive for *vtrB* expression). In the WT strain, the S transcript of ~700 nt from the *vtrB* promoter, derived from primary activation, appeared 15 min post-induction. The ~4,000 nt L and ~2,000 nt M transcripts generated by secondary activation via read-through transcription of the operon upstream of *vtrB* were detected 30 min after induction. In the Δ*tsrA* strain, increased levels of the S transcript were observed at 15 min post-induction and L/M transcripts from 30 min post-induction. We also measured *vtrB* transcript levels by quantitative real-time PCR (qRT-PCR) targeting the 5′-untranslated region of the *vtrB* gene to quantitatively compare *vtrB* expression in each strain. The *vtrB* transcript was significantly increased in the ∆*tsrA* strain compared to that in the WT strain 15 min post-induction (Fig. 2B).

To further determine whether TsrA affects primary or secondary activation of *vtrB* gene expression, we tested the effect of *tsrA* deletion on *vtrB* gene expression over time by northern blotting with the *vtrB*-deleted strain (∆*vtrB*) lacking secondary activation, as a background (Fig. 2A). In the ∆*vtrB* strain, a ~200 nt *vtrB* transcript with a deletion in the *vtrB* coding sequence was detected 15 min after induction. The *tsrA*-deleted strain derived from the ∆*vtrB* strain (∆*vtrB* ∆*tsrA*) showed increased *vtrB* transcript levels. Consistent with this, qRT-PCR analysis also indicated the upregulation of *vtrB* transcripts in the ∆*vtrB* ∆*tsrA* strain compared to that in the ∆*vtrB* strain (Fig. 2B). Collectively, these results indicate that TsrA affects *vtrB* expression during primary activation.

### TsrA negatively affects T3SS2-dependent virulence

**Fig 3:**
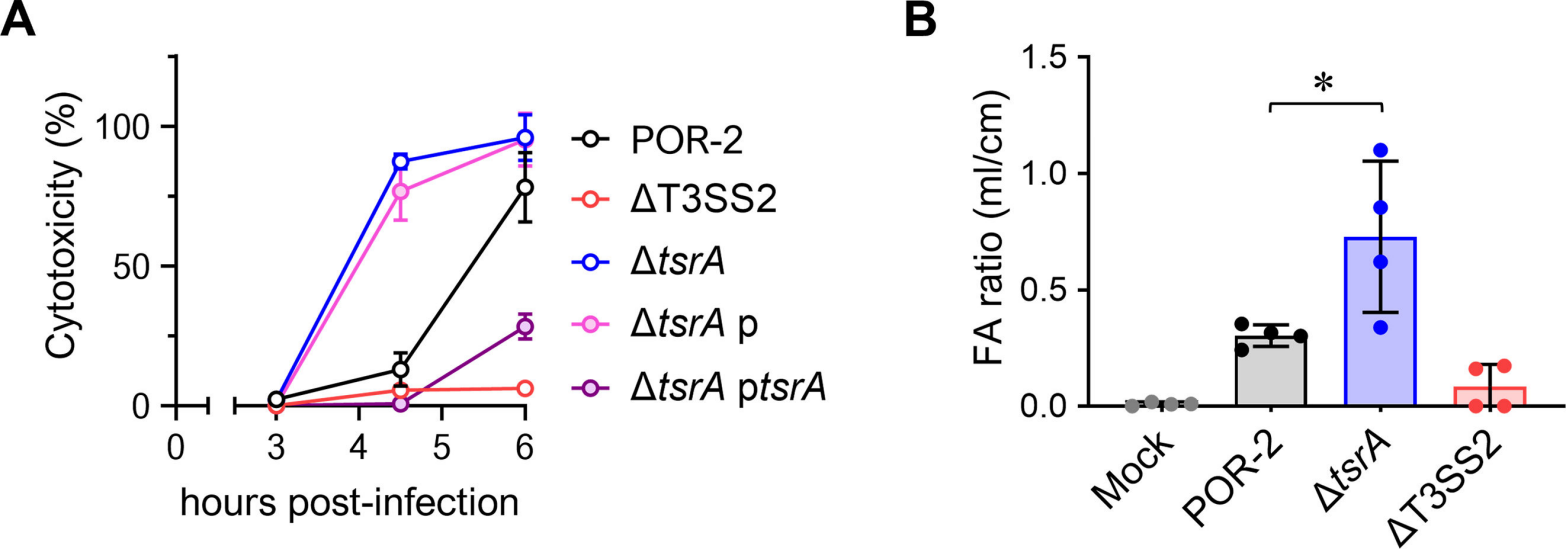
TsrA negatively affects the T3SS2-dependent virulence. (**A**) Time-course analysis of the cytotoxicity of Caco-2 cells infected with the indicated *V. parahaemolyticus* strains at a multiplicity of infection of 10. Cytotoxicity was evaluated by measuring lactate dehydrogenase release. Data are represented as mean ± SD (*n* = 3). (**B**) Fluid accumulation induced by the indicated *V. parahaemolyticus* strains was analyzed using the rabbit ileal loop model. Each ligated loop was infected with bacteria (POR2 and Δ*tsrA*: 10^8^ CFU; ΔT3SS2: 10^9^ CFU) or was uninfected, and fluid accumulation in the loop was evaluated 16 h post-infection. The fluid accumulation (FA) ratio represents the amount of accumulated fluid (mL) per length (cm) of the ligated small intestine. Data are represented as mean ± SD (*n* = 4). *, *P* < 0.01 by student’s *t*-test.

Given that T3SS2 plays a crucial role in the pathogenicity of *V. parahaemolyticus*, including cytotoxicity and enterotoxicity (18), we investigated whether TsrA affects T3SS2-mediated virulence. *V. parahaemolyticus* exhibits T3SS2-dependent cytotoxicity in human colon adenocarcinoma Caco-2 cells. To exclude cytotoxicity depending on other factors, such as type III secretion system 1 (T3SS1) and thermostable direct hemolysin (TDH), a T3SS1- and TDH-defective strain POR-2 was used as a background. Caco-2 cells were infected with *V. parahaemolyticus* POR-2 strain or its derivative, the *tsrA*-deleted strain (POR-2 ∆*tsrA*), and cytotoxicity was evaluated (Fig. 3A). Notably, POR-2 ∆*tsrA* was significantly more cytotoxic than the parental POR-2 strain, causing complete cell death 4.5 h after infection, and this higher cytotoxicity was attenuated by plasmid-mediated complementation of *tsrA*.

Diarrhea-inducing activity is an important virulence trait of *V. parahaemolyticus* mediated by T3SS2. Therefore, we investigated the effect of TsrA on the diarrhea-inducing activity of *V. parahaemolyticus* in the rabbit ileal loop model, in which *V. parahaemolyticus* strains were inoculated into the ligated ileal loops of rabbits, and fluid accumulation (FA) in each loop was measured 16 h after infection. The ∆*tsrA* strain induced significantly increased fluid accumulation in rabbit ileal loops compared to the parental strain POR-2 (Fig. 3B). These results indicate that TsrA suppresses T3SS2-mediated pathogenicity.

### TsrA interacts with H-NS

**Fig 4:**
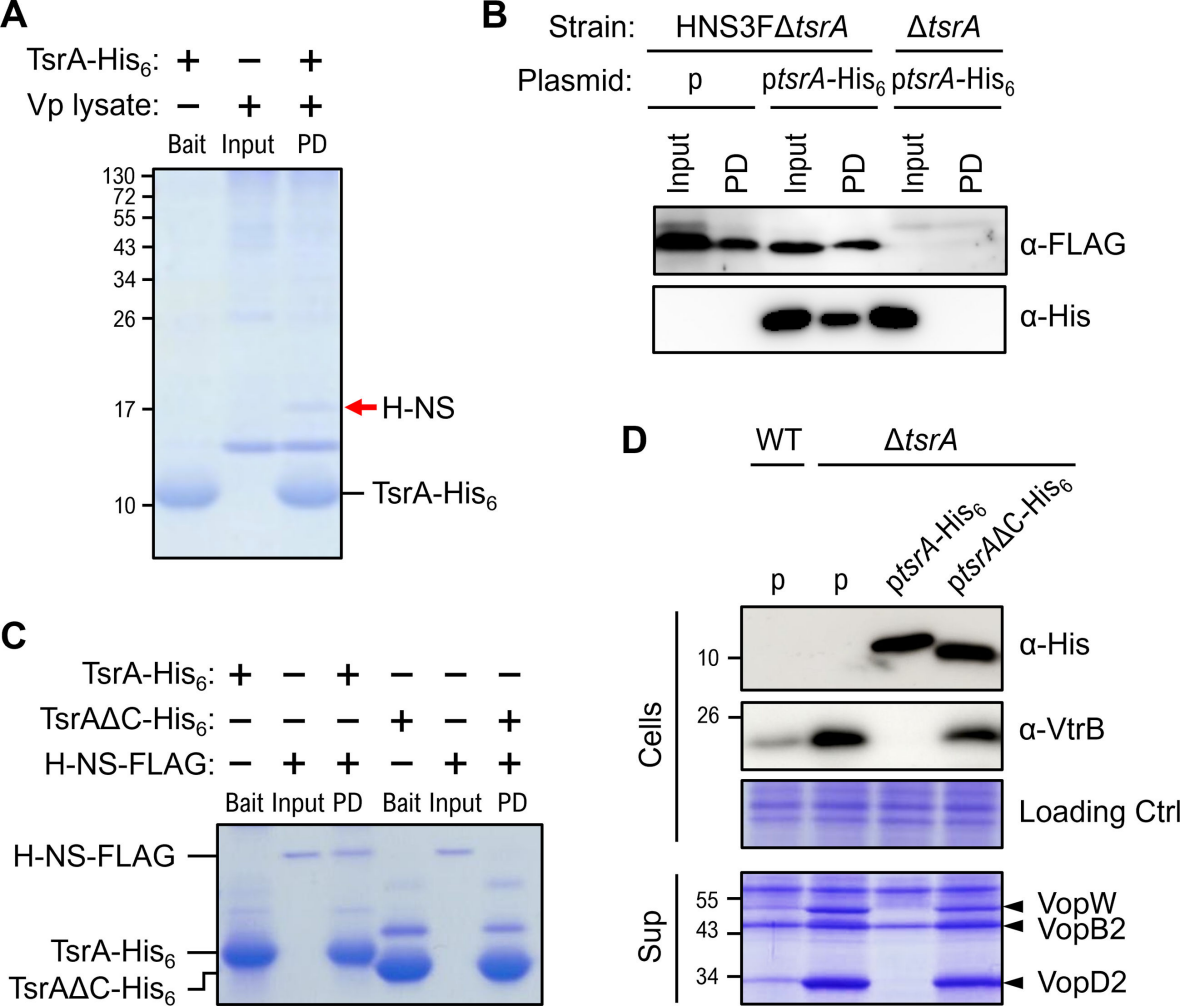
TsrA interacts with H-NS. (**A**) TsrA-interacting proteins were purified from *V. parahaemolyticus* lysates using a TsrA-His_6_-immobilized Ni-NTA column. Bound proteins were separated by SDS-PAGE and visualized by CBB staining. The arrow indicates H-NS. (**B**) FLAG co-immunoprecipitation from lysates of the HNS-3F Δ*tsrA* strain harboring either p*tsrA*–His_6_ (encoding TsrA-His_6_) or an empty vector and from the Δ*tsrA* strain harboring p*tsrA*–His_6_. Bound proteins were eluted and analyzed by immunoblotting using anti-FLAG or anti-His antibodies. PD, pull down. (**C**) Binding of H-NS to TsrA or TsrAΔC. TsrA-His_6_ or TsrAΔC-His_6_ was immobilized on a Ni-NTA column, and purified H-NS-FLAG was applied. The eluates were analyzed by SDS-PAGE and visualized by CBB staining. (**D**) The C-terminal region of TsrA is required for the repression of VtrB production and T3SS2 secretion. *V. parahaemolyticus* WT and Δ*tsrA* strains carrying an empty vector (p), or Δ*tsrA* complemented with p*tsrA*-His_6_ or p*tsrA*ΔC-His_6_ were grown in Luria–Bertani (LB) medium at 37°C to an OD_600_ of 1.8. Cell lysates (Cells) were subjected to immunoblotting with the indicated antibodies, with CBB-stained proteins serving as the loading control. Culture supernatants (Sup) were analyzed by SDS-PAGE and CBB staining. T3SS2-secreted proteins (VopW, VopB2, and VopD2) are marked by arrowheads.

We then aimed to determine how TsrA represses *vtrB* expression. Given that TsrA does not contain any recognizable DNA-binding motif for the direct regulation of transcription based on sequence analysis, we hypothesized that TsrA interacts with other factors to repress *vtrB* transcription. To identify *V. parahaemolyticus* proteins that interact with TsrA, C-terminally 6 × His-tagged TsrA (TsrA-His_6_) expressed in *Escherichia coli* was immobilized on Ni-NTA beads and incubated with *V. parahaemolyticus* lysates. Proteins bound to TsrA were separated by SDS-PAGE, followed by Coomassie Brilliant Blue (CBB) staining, and a protein of ~17 kDa was found to be specifically associated with TsrA (Fig. 4A). The protein band was subjected to liquid chromatography-tandem mass spectrometry (LC-MS/MS) analysis and was subsequently identified as H-NS. We also subjected purified 3 × FLAG-tagged H-NS protein (H-NS-FLAG) to Ni-NTA beads immobilized with TsrA-His_6_, which demonstrated direct binding between TsrA and H-NS (Fig. S2). Furthermore, co-immunoprecipitation from the lysate of a *V. parahaemolyticus* strain co-expressing H-NS-FLAG and TsrA-His_6_ revealed that TsrA co-precipitated with H-NS, confirming their *in vivo* interaction (Fig. 4B). These results indicate that TsrA interacts with H-NS.

**Fig 5:**
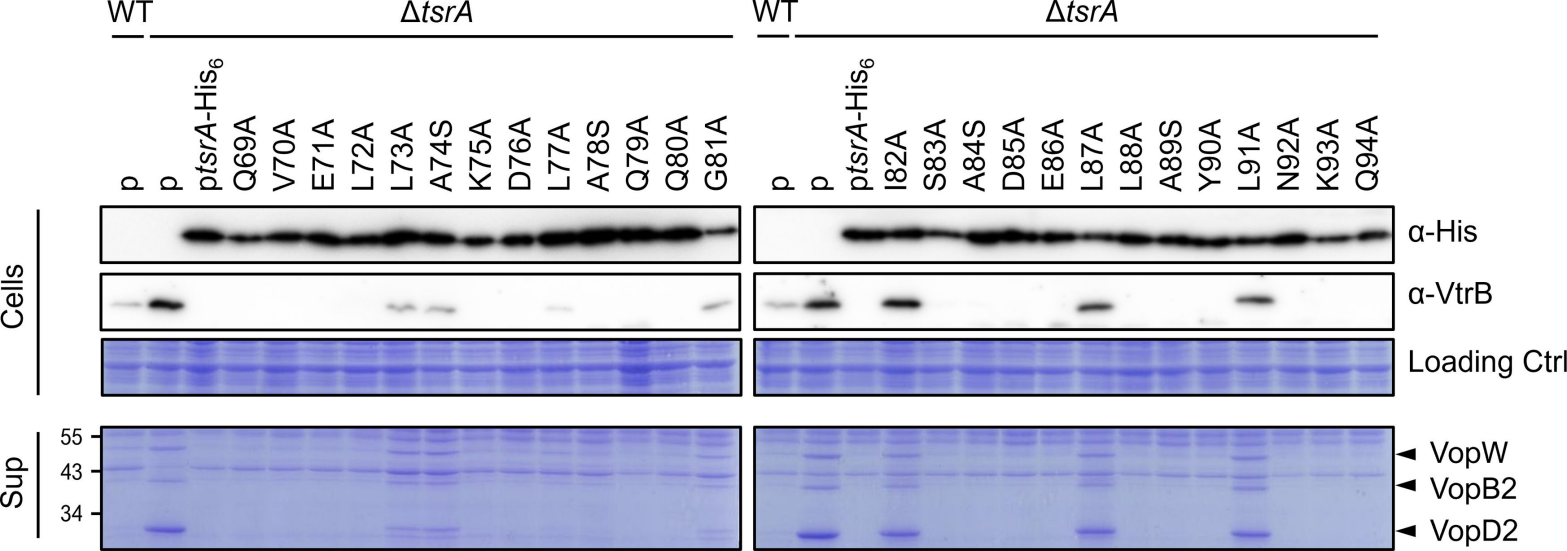
Mutational analysis of the C-terminal region of TsrA. *V. parahaemolyticus* WT harboring an empty vector (p), and the Δ*tsrA* strain carrying either an empty vector, p*tsrA*-His_6_, or p*tsrA*-His_6_ derivatives containing Ala/Ser substitutions in the C-terminal region (residues 69–94) were grown in Luria–Bertani (LB) medium at 37°C to an OD600 of 1.8. Culture supernatants (Sup) were analyzed by SDS-PAGE, followed by CBB staining. Whole-cell lysates (cells) were subjected to immunoblotting with anti-VtrB and anti-His antibodies, with CBB-stained proteins serving as a loading control. An asterisk indicates a nonspecific background band.

To gain insight into the structural basis of this interaction, we performed structural modeling using AlphaFold3, which suggested that the C-terminal region of TsrA interacts with the N-terminal dimerization domain of H-NS (Fig. S3). To determine whether the C-terminal region of TsrA is required for binding to H-NS, we constructed a truncated TsrA mutant lacking the C-terminal region (residues 1–68, TsrA∆C) and evaluated its binding activity. TsrA-His_6_ or TsrA∆C-His_6_ protein expressed in *E. coli* was immobilized on Ni-NTA beads, and its interaction with purified H-NS-FLAG was examined. In contrast to full-length TsrA, TsrA∆C did not interact with H-NS (Fig. 4C). Furthermore, complementation of the Δ*tsrA* strain with *tsrA*-His_6_ (Δ*tsrA* p*tsrA-*His_6_) abolished the increased expression of VtrB and hypersecretion of T3SS2, whereas the expression of *tsrA*∆C-His_6_ in the Δ*tsrA* strain (Δ*tsrA* p*tsrA*∆C*-*His_6_) did not restore these phenotypes (Fig. 4D). To further characterize the interaction between TsrA and H-NS, we performed Ala/Ser scanning mutagenesis of the C-terminal region of TsrA (residues 69–94). A series of p*tsrA-*His_6_ derivatives were expressed in the ∆*tsrA* strain, and VtrB production and VopD2 secretion were assessed. Among the 26 mutants, I82A, L87A, and L91A showed higher levels of VtrB production and VopD2 secretion than wild-type TsrA (Fig. 5), indicating that these residues contribute to the regulatory activity of TsrA, possibly by mediating its interaction with H-NS. This notion is supported by the AlphaFold3 structural model, in which these residues were predicted to be positioned at the interface with H-NS (Fig. S3). Collectively, these results indicate that the C-terminal region of TsrA mediates its interaction with H-NS, which is critical for negative regulation of T3SS2.

### TsrA-mediated T3SS2 repression requires H-NS

To examine the functional consequence of the TsrA–H-NS interaction in the regulation of T3SS2, we constructed a *tsrA*- and *hns*-deleted mutant (Δ*tsrA* Δ*hns*) and analyzed VtrB production and VopD2 secretion. As reported previously (5), deletion of *hns* alone did not lead to increased VtrB protein levels or VopD2 secretion compared with the WT strain (Fig. S4A), in contrast to the phenotype observed in the *tsrA* mutant. The Δ*tsrA* Δ*hns* mutant displayed VtrB protein levels and VopD2 secretion comparable to those of the Δ*hns* mutant. Complementation of the double mutant with *hns* restored the increased VtrB protein levels and VopD2 secretion, whereas complementation with *tsrA* did not, resulting in a phenotype similar to that of Δ*hns*. These results indicate that TsrA-mediated repression of T3SS2 is dependent on H-NS.

## DISCUSSION

Pathogenic bacteria rely on precise regulation of virulence gene expression to ensure survival and adaptation in diverse environments. In *V. parahaemolyticus*, T3SS2 is a major virulence determinant, and its activity is governed by regulatory circuits that integrate environmental cues (5, 19). Here, we identified TsrA as a negative regulator of T3SS2 gene expression, thereby uncovering a previously unrecognized layer in the regulatory network of *V. parahaemolyticus*. This regulation is achieved by modulating the expression of VtrB, a transcriptional activator essential for T3SS2 expression. The transcription of *vtrB* is primarily driven by the VtrA/VtrC complex and secondarily amplified through read-through transcription from the upstream *VPA1356–VPA1349* operon (11–13). Our findings indicate that the negative effect of TsrA acts specifically on the primary activation step of *vtrB* expression (Fig. 2), without altering the expression of the initial activators (Fig. 1C). This mechanism likely functions to restrict *vtrB* expression levels, suggesting that TsrA plays a crucial role in controlling the amplification of *vtrB* expression.

The absence of a putative DNA-binding domain in TsrA suggests that its function is mediated not by direct DNA recognition, but by interactions with other regulatory factors. We found that TsrA interacts with H-NS (Fig. 4), a nucleoid-associated protein that often cooperates with co-regulators to modulate repression strength and gene specificity (20, 21). Our results suggest that TsrA may act as an H-NS co-regulator in *V. parahaemolyticus*, thereby expanding the repertoire of known H-NS-associated modulators. This is reminiscent of observations in *V. cholerae*, where TsrA is implicated in a functional association with H-NS, with a structural model predicting its interaction (16, 17). Nevertheless, to our knowledge, our data provide the first experimental evidence of TsrA-H-NS interactions. Given that H-NS regulates *vtrB* expression through direct binding to the *vtrB* promoter (5), it is reasonable that TsrA also acts on the primary activation of *vtrB* transcription through H-NS. Structural modeling using AlphaFold3 predicted that the C-terminal region of TsrA interacts with the N-terminal oligomerization domain of H-NS (Fig. S3). Notably, a TsrA variant lacking the C-terminal region (residues 69–94) failed to interact with H-NS and lost its ability to repress *vtrB* expression and T3SS2 secretion (Fig. 4C and D). In addition, our Ala/Ser scanning analysis identified several residues in the C-terminal region of TsrA that are important for repressive activity. These residues are hydrophobic amino acids and are predicted, in the AlphaFold3 model, to interact with hydrophobic residues within the oligomerization site in the N-terminal domain of H-NS (site 2), potentially forming an interlocking between TsrA and H-NS through hydrophobic interactions (Fig. 5; Fig. S3B). These observations suggest that the interaction with H-NS underlies the regulatory activity of TsrA, and TsrA may mimic or complement H-NS structural motifs to facilitate complex formation. Several studies have shown that H-NS exerts its repressive activity through its ability to oligomerize along DNA, forming linear and/or bridged nucleoprotein filaments (22). These filaments prevent RNA polymerase (RNAP) from initiating transcription at promoters, halt its elongation, and trap it within DNA loops (23). Among well-known H-NS co-regulators, such as StpA and Hha-like proteins in *Escherichia coli* and *Salmonella enterica* (24, 25), TsrA shares a feature with Hha-like proteins in that it lacks a DNA-binding domain. Hha binds symmetrically to the dimerization site in the N-terminal domain of H-NS (site 1) at a 1:1 ratio, exposing a positively charged surface that enhances H-NS binding to DNA (20, 26). The interaction between Hha and H-NS stimulates DNA-DNA bridging activity and increases RNAP pausing (21). In contrast to Hha, our predicted model suggests that TsrA dimerizes via its N-terminal region, with each C-terminal domain engaging the oligomerization site 2 of H-NS, bridging two H-NS monomers without creating an exposed positively charged surface. Here, when an N-terminal deletion variant of His-tagged TsrA (1–68) was expressed from a plasmid in *V. parahaemolyticus*, no expression was detected (data not shown), suggesting that the predicted N-terminal dimerization domain may contribute to the stability of TsrA. These observations suggest that TsrA operates mechanistically differently from Hha, likely contributing to the stabilization of H-NS nucleoprotein filament complexes and modulating their activity rather than directly facilitating H-NS filament–DNA binding. A deeper mechanistic understanding of how TsrA modulates H-NS activity warrants further exploration in future studies, such as ChIP-seq, to determine whether and how TsrA influences H-NS binding dynamics or higher-order nucleoprotein architecture.

The distribution of Hha-like proteins is restricted to *Enterobacteriaceae* (27), whereas TsrA is found in *Vibrionaceae* (14, 16). This evolutionary distinction may reflect the mechanistic differences between TsrA and Hha as H-NS co-regulators of gene expression. The notion that TsrA functions as an H-NS co-regulator is also supported by our observation in the H-NS–TsrA double mutant (Fig. S4). Interestingly, under the permissive condition for T3SS2 expression, the *hns* mutant does not exhibit increased expression of *vtrB* or T3SS2 genes relative to the wild-type strain (5), unlike the *tsrA* mutant. Nevertheless, deletion of both *tsrA* and *hns* abolished the upregulation of *vtrB* expression and T3SS2 secretion observed in the *tsrA* single mutant. These findings indicate that TsrA-mediated T3SS2 repression is dependent on H-NS. Because deletion of *hns* is known to cause global transcriptional changes (28), the *vtrB* expression level observed in the Δ*hns* mutant may reflect indirect effects resulting from altered regulatory networks, thereby masking the H-NS-dependent repression of T3SS2 genes that would otherwise be observed in the absence of TsrA.

Our transcriptomic analysis demonstrated that TsrA affects not only T3SS-related genes but also genes beyond the Vp-PAI (Table S1). The majority of these genes are encompassed within the H-NS regulon, including polar flagella-related genes, the putative fimbrial regulatory protein (*vp0377*), the putative F-related protein (*vpa0548*), the chemotaxis protein (*vpa0842*), and several hypothetical proteins (*vp0051* and *vp0384*). This feature is analogous to that observed in the *tsrA* mutant in *V. cholerae*, where the effects of TsrA are largely confined to a subset of genes within the H-NS regulon (14, 16), supporting a model in which TsrA acts in concert with H-NS to selectively modulate H-NS-regulated genes.

Collectively, our study identifies TsrA as a novel virulence repressor in *V. parahaemolyticus*, revealing a mechanism by which this small protein restricts T3SS2 activation through modulating *vtrB* transcription initiation. Moreover, we provide experimental evidence that TsrA interacts with H-NS, highlighting TsrA as an H-NS co-regulator (Fig. S4B). Although further investigation is warranted to elucidate how TsrA integrates into H-NS-mediated silencing, our findings not only advance the understanding of how virulence regulation is fine-tuned in *V. parahaemolyticus* but also shed light on the evolutionary strategies underlying the xenogeneic silencing of horizontally acquired genes.

## MATERIALS AND METHODS

### Bacterial strains, primers, plasmids, and growth conditions

The clinical isolate *Vibrio parahaemolyticus* RIMD2210633 (29) was used as the wild-type strain. *E. coli* DH5α and BW19851 were used for DNA manipulation. All bacterial strains and plasmids used in this study are listed in Tables S2 to S4. The primers used are listed in Table S5. A four-primer PCR technique was used to engineer the deletion mutants, as described previously (30). Plasmids encoding TsrA–His_6_ derivatives, in which residues 69–94 in the C-terminal region were individually substituted with alanine or serine if the original residue was alanine, were constructed by site-directed mutagenesis using the appropriate primer pairs. *V. parahaemolyticus* strains were grown at 37°C in Luria–Bertani (LB) medium (Lennox: tryptone, 1%; yeast extract, 0.5%; NaCl, 0.5%) with shaking at 200 rpm. Appropriate antibiotics were added to grow the plasmid-carrying strains: 50 μg/mL kanamycin for pET28a and pE-SUMO-Kan, and 15 μg/mL chloramphenicol for pSA19CP plasmid backgrounds.

### Protein sample preparation and analysis

*V. parahaemolyticus* strains were grown in LB medium at 37°C to an optical density at 600 nm (OD_600_) of 1.8. The culture was centrifuged at 15,000 × *g* for 5 min to separate the pellets containing the bacterial cells from the supernatants containing the secreted proteins. The pellets were solubilized in SDS-PAGE sample buffer, followed by sonication and denaturation at 95°C for 5 min, and used as whole-cell lysates. Secreted proteins were precipitated from the supernatants with ice-cold 10% (vol/vol) trichloroacetic acid (TCA) on ice for 2 h, followed by centrifugation at 25,000 × *g* for 30 min. The resulting pellets were rinsed with cold acetone, solubilized in SDS-PAGE sample buffer, and denatured at 95°C for 5 min. Proteins were then separated by SDS-PAGE and either visualized by CBB staining or transferred to polyvinylidene difluoride membranes for immunoblotting. The membranes were probed with primary antibodies against the proteins of interest. Anti-VtrA, anti-VtrB, and anti-VopD2 polyclonal antibodies were described elsewhere (10, 31). Monoclonal antibodies against anti-His-tag (clone OGHis, Medical and Biological Laboratories) and anti-FLAG (clone M2, Sigma-Aldrich) were purchased from commercial suppliers. The membranes were then probed with horseradish peroxidase (HRP)-conjugated goat anti-rabbit antibodies (code: 62–1820, Invitrogen) or HRP-conjugated rabbit anti-mouse antibodies (code: 61–6520, Invitrogen). The blots were developed using the ECL Prime Western Blotting Detection Reagent (GE Healthcare). Whole-cell lysate proteins with CBB staining of each sample were used as loading controls for immunoblotting. To detect TsrA–His_6_ and its derivatives, whole-cell lysates were further concentrated by TCA precipitation, or the proteins were enriched from *V. parahaemolyticus* lysates using Ni-NTA pull-down as follows. *V. parahaemolyticus* strains were grown in LB medium at 37°C to OD_600_ of 1.8. Bacterial cells were harvested by centrifugation at 15,000 × *g* for 5 min and lysed by sonication (10 s pulse, 30 s interval on ice, and 10 cycles), repeated seven times, in Ni-NTA running buffer (25 mM Tris-HCl, 50 mM NaCl, pH 8.0) containing a protease inhibitor cocktail (P8849, Sigma-Aldrich), 40 U/mL benzonase (Sigma-Aldrich), and 1 mg/mL lysozyme (Sigma-Aldrich). The lysate was incubated with Ni-NTA His·Bind beads (Novagen) on a rotary shaker for 2 h at 4°C, to capture His-tagged proteins. The beads were washed five times with Ni-NTA wash buffer (25 mM Tris-HCl, 50 mM NaCl, 60 mM imidazole, pH 8.0), and bound proteins were eluted with Ni-NTA elution buffer (25 mM Tris-HCl, 200 mM NaCl, 500 mM imidazole, pH 8.0). The eluates were analyzed by immunoblotting using anti-His-tag antibody.

### Cell culture and cytotoxicity assay

Caco-2 cells (ECACC 86010202) supplied by the European Collection of Authenticated Cell Cultures were maintained in Dulbecco’s modified Eagle’s medium (DMEM) supplemented with 10% fetal bovine serum and 100 μg/mL gentamicin at 37°C under 5% CO_2_. Cytotoxicity assays were performed as previously described (32), with minor modifications. Briefly, 2 × 10^4^ Caco-2 cells were seeded into each well of a 96-well plate and incubated for 48 h. The cells were washed twice with phosphate-buffered saline, and the medium was replaced with fresh phenol red-free DMEM. The cells were then infected with each *V. parahaemolyticus* strain at a multiplicity of infection of 10. Cytotoxicity was assessed by measuring the release of lactate dehydrogenase (LDH) into the culture supernatants using a Cytotoxicity LDH Assay Kit-WST (Dojindo) according to the manufacturer’s instructions. The percentage of cytotoxicity was calculated as follows: [OD_490_ of experimental release – OD_490_ of spontaneous release]/[OD_490_ of maximum release – OD_490_ of spontaneous release] × 100. Spontaneous release refers to the amount of LDH released from uninfected cells, whereas maximum release refers to the total LDH released after complete lysis of uninfected cells by detergent.

### Rabbit ileal loop assay

Rabbit ileal loop tests were performed as previously described (32). Briefly, *V. parahaemolyticus* strains were grown in LB medium. The cells were harvested by centrifugation and suspended in LB medium. One milliliter of the bacterial suspension (10^9^ colony-forming units) was injected into the ligated ileal loops of a 1.5–2 kg female New Zealand White rabbit (loop length ~8 cm). Fluid accumulation in each loop was measured 16 h after inoculation. The FA ratio was calculated as the volume of accumulated fluid (milliliter) per length of the ligated rabbit small intestine (centimeter).

### RNA sequencing

Total RNA was extracted from *V. parahaemolyticus* as described previously (5). Briefly, *V. parahaemolyticus* cells were grown in LB containing 0.5% NaCl at 37°C to an OD_600_ of ~1. RNAprotect Bacterial Reagent (Qiagen) was added to the culture to stabilize RNA, and total RNA was extracted using the RNeasy Mini Kit (Qiagen) with DNase I treatment, according to the manufacturer’s instructions. Purified RNA samples were analyzed using PCR to confirm the absence of DNA contamination. The concentration of each sample was measured using a NanoDrop ND-1000 spectrophotometer (Thermo Fisher).

RNA libraries were prepared using the TruSeq Stranded Total RNA with RiboZero Plus Kit (Illumina). Whole-transcriptome sequencing was performed using an Illumina NovaSeq 6000 platform in the 151 bp single-end mode. Sequence reads were mapped to the *V. parahaemolyticus* reference genome (GCF_000196095.1) using Bowtie2 v2.4.4 (31), and the gene counts were obtained using HTSeq v0.11.1 (33). The read counts data were uploaded onto iDEP 2.20.4 (34), and differential gene expression was analyzed using the DESeq2 option.

### Northern blot analysis and qRT-PCR

*V. parahaemolyticus* strains were grown in LB medium to an OD_600_ of 0.8 at 25°C, a non-permissive condition for *vtrB* expression, and then shifted to 37°C to induce *vtrB* transcription. RNA was extracted at 0, 15, 30, and 60 min after induction using the hot-acidic phenol method (35). Northern blotting was performed as previously described (13). Briefly, total RNA (2.5 μg) was heated at 60°C and separated by electrophoresis on a 1.2% agarose gel containing formaldehyde alongside Dyna Marker (Prestain Marker for RNA High; BioDynamics Laboratory Inc). The RNA was then transferred overnight onto a positively charged nylon membrane (Roche) using 20 × SSC buffer (3 M NaCl, 0.3 M sodium citrate dehydrate) and cross-linked by UV irradiation (1,200 × 100 μJ/cm²). Hybridization was carried out at 50°C for 4 h in DIG Easy Hyb buffer (Roche) using the DIG-labeled P*_vtrB_* probe (13). After hybridization, the membrane was washed with a high-stringency buffer (0.1 × SSC, 0.1% SDS), blocked for 30 min in blocking solution (Roche), and incubated for 1 h with anti-digoxigenin-alkaline phosphatase (anti-DIG-AP; Roche). The blots were developed using CDP-STAR chemiluminescent substrate (Roche) and visualized using an Amersham ImageQuant 800 system (Cytiva). Ribosomal RNA was stained with GelRed (Biotium, Hayward, CA, USA) and visualized by UV irradiation as the loading control. qRT-PCR was performed as previously described (13) using SYBR Green RNA-direct Real-time PCR Master Mix (Toyobo) on a QuantStudio 5 real-time PCR system (Thermo Fisher). Relative mRNA transcript levels were calculated using the threshold cycle (2^−ΔΔCT^) method and normalized to the housekeeping gene *recA*.

### Purification of TsrA-interacting proteins

Protein expression was performed as previously described (5). Briefly, the gene for C-terminally 6 × His-tagged TsrA (TsrA-His_6_) or its derivative was inserted into the pET28a expression plasmid (Novagen), yielding TsrA-His_6_ constructs. *E. coli* BL21 (DE3) cells carrying these plasmids were grown in LB medium with 50 μg/mL kanamycin at 37°C to an OD_600_ of ~0.6. Protein expression was induced by adding 1 mM isopropyl-1-thio-β-D-galactopyranoside, followed by further incubation at 37°C for 4 h. Bacterial cells were harvested by centrifugation at 10,000 × *g* for 15 min and lysed in CelLytic B cell lysis reagent (Sigma-Aldrich) containing a protease inhibitor cocktail (P8849, Sigma-Aldrich), 40 U/mL benzonase (Sigma-Aldrich), and 1 mg/mL lysozyme (Sigma-Aldrich), followed by sonication (10 s pulse, 30 s interval on ice, and 10 cycles), repeated seven times. The cell lysates were centrifuged at 10,000 × *g* for 15 min, and the resulting supernatants, including solubilized proteins, were filtered through a 0.22 μm filter to remove insolubilized proteins and unbroken cells. Each 6 × His-tagged protein was immobilized on Ni-NTA His·Bind beads (Novagen) packed in a column, according to the manufacturer’s protocol. For purification of TsrA-interacting proteins, *V. parahaemolyticus* ∆*tsrA* strain grown in LB medium was lysed in Ni-NTA running buffer (25 mM Tris-HCl, 50 mM NaCl, pH 8.0) containing a protease inhibitor cocktail (P8849, Sigma-Aldrich), 40 U/mL benzonase (Sigma-Aldrich), and 1 mg/mL lysozyme (Sigma-Aldrich), and the lysate was incubated with TsrA-His_6_-immobilized beads on a rotary shaker for 2 h at 4°C. The beads were washed five times with Ni-NTA wash buffer (25 mM Tris-HCl, 50 mM NaCl, 60 mM imidazole, pH 8.0), and bound proteins were eluted from the beads with Ni-NTA elution buffer (25 mM Tris-HCl, 200 mM NaCl, 500 mM imidazole, pH 8.0). The eluates were subjected to SDS-PAGE followed by CBB staining or LC-MS/MS analysis with a timsTOF Pro (Bruker) at the Core Instrumentation Facility, Research Institute for Microbial Diseases, The University of Osaka, as described previously (5). Proteins were identified by searching the database using Mascot (Matrix Science).

### Direct interaction between TsrA and H-NS

For purification of C-terminally 3 × FLAG-tagged H-NS (H-NS-FLAG), the gene encoding H-NS-FLAG was inserted into pE-SUMO-Kan (LifeSensors) to generate the 6 × His-SUMO-tagged H-NS-FLAG. The C-terminal region of *ULP1* encoding SUMO protease (403–621 aa, Ulp1C) was cloned into pET28a to yield 6 × His-U1p1C. *E. coli* BL21 (DE3) or Rosetta-gami 2 (DE3; Novagen) cells harboring the respective plasmids were grown in LB medium with 50 μg/mL kanamycin to an OD_600_ of 0.6. Protein expression was induced by adding 0.1 mM isopropyl-1-thio-β-D-galactopyranoside (Sigma), followed by incubation for 4 h at 37°C for 6 × His-SUMO-tagged H-NS-FLAG or for 16 h at 20°C for 6 × His-Ulp1C. Expressed 6 × His-SUMO-tagged H-NS-FLAG and 6 × His-Ulp1C were purified using Ni-NTA His·Bind beads (Novagen) according to the manufacturer’s instructions. The 6 × His-SUMO-tag was cleaved from the purified 6 × His-SUMO-tagged H-NS-FLAG by 6 × His-tagged Ulp1C in a digestion buffer (20 mM Tris-HCl, 150 mM NaCl, 1 mM dithiothreitol, pH 7.6) at 30°C for 24 h, and then passed through TALON Metal Affinity Resin (Takara) columns to remove the released His-SUMO tag, undigested fusion proteins, and 6 × His-Ulp1C, yielding the H-NS-FLAG protein. The purity of the purified protein was confirmed by SDS–PAGE followed by CBB staining. Protein concentrations were measured using a Nanodrop ND-1000. To examine the direct interaction between TsrA and H-NS, Ni-NTA His·Bind beads immobilized with TsrA-His_6_ or control beads were incubated with 25 μM purified H-NS-FLAG protein or bovine serum albumin on a rotary shaker for 2 h at 4°C. The beads were washed five times with the washing buffer and then eluted with the elution buffer. The eluates were subjected to SDS–PAGE, followed by CBB staining or immunoblotting with anti-His and anti-FLAG antibodies.

### Co-immunoprecipitation

To construct a *V. parahaemolyticus* strain expressing TsrA-His_6_ and H-NS-FLAG, the *tsrA* gene was deleted from the HNS-3F strain, which chromosomally expresses H-NS-FLAG (5), resulting in the HNS-3F∆*tsrA* strain. The plasmid pSA19CP encoding *tsrA*-His_6_ (p*tsrA*-His_6_), its derivatives, or the empty vector was introduced into this strain, yielding strains expressing TsrA-His_6_, its derivatives, or harboring the empty vector as a control strain. These strains, along with the ∆*tsrA* strain harboring p*tsrA-*His_6_, were grown in LB medium and lysed in Ni-NTA running buffer (25 mM Tris-HCl, 50 mM NaCl, pH 8.0) containing a protease inhibitor cocktail (P8849, Sigma-Aldrich), 40 U/mL benzonase (Sigma-Aldrich), and 1 mg/mL lysozyme (Sigma-Aldrich). The lysates were subjected to co-immunoprecipitation using anti-FLAG M2 beads (Sigma-Aldrich). The beads were washed five times with FLAG washing buffer (20 mM Tris-HCl, 200 mM KCl, 5 mM MgCl_2_, 10% glycerol, pH 8.0) and eluted using 2 × SDS-PAGE sample buffer. The eluates were analyzed by immunoblotting with anti-His and anti-FLAG antibodies.

### Structural modeling

Models of the H-NS and TsrA complexes were generated using AlphaFold3 (https://alphafoldserver.com) (36) with *Vibrio parahaemolyticus* TsrA (uniprot: Q87KF9) and H-NS (uniprot: Q87QL6) protein sequences as inputs. The resulting structural models were visualized using ChimeraX (37).

## Data Availability

The RNA-seq datasets in this study were deposited in the NCBI Sequence Read Archive (SRA) under the accession number PRJNA1357053. The mass spectrometry datasets generated in this study were deposited in the jPOST repository (ProteomeXchange accession PXD073088; jPOST ID JPST004329).

## SUPPLEMENTAL MATERIAL

Supplemental figures jb.00556-25-s0001.pdf Figures S1–S4.

Table S1 jb.00556-25-s0002.xlsx RNA-seq dataset.

Tables S2 to S5 jb.00556-25-s0003.pdf Tables S2–S5.

ASM does not own the copyrights to Supplemental Material that may be linked to, or accessed through, an article. The authors have granted ASM a non-exclusive, world-wide license to publish the Supplemental Material files. Please contact the corresponding author directly for reuse.
