# Supplementary material for: TsrA modulates type III secretion system 2 expression as a co-regulator of H-NS in Vibrio parahaemolyticus
Source: J Bacteriol. 2026 May 28;208(6):e00556-25. doi: 10.1128/jb.00556-25 (PMC13277298; doi:10.1128/jb.00556-25)
Supplement: Supplemental figures — Figures S1–S4. [file jb.00556-25-s0001.pdf]

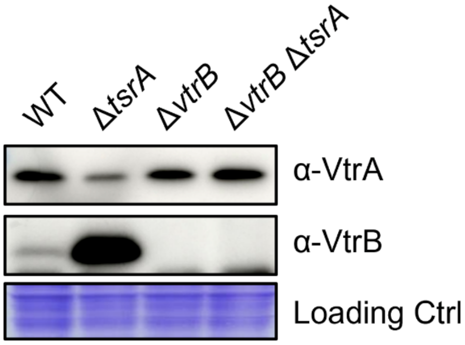

Fig S1. VtrA reduction in Δ*tsrA* is mediated by VtrB

V. parahaemolyticus strains WT, Δ*tsrA*, Δ*vtrB*, and Δ*vtrB* Δ*tsrA* were grown in LB medium at 37°C to an OD_{600} of 1.8. Bacterial cell lysates were analyzed by immunoblotting for VtrA and VtrB, with proteins visualized by CBB staining serving as a loading control.

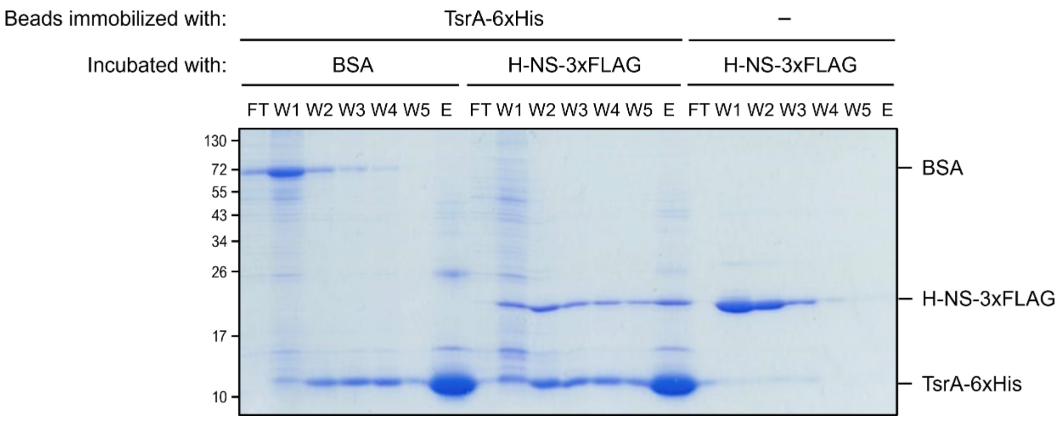

Fig S2. TsrA directly interacts with H-NS

Ni-NTA beads immobilized with TsrA-6×His protein or control beads were incubated with purified H-NS-3×FLAG protein or bovine serum albumin (BSA). The eluates were analyzed by SDS-PAGE, followed by CBB staining.

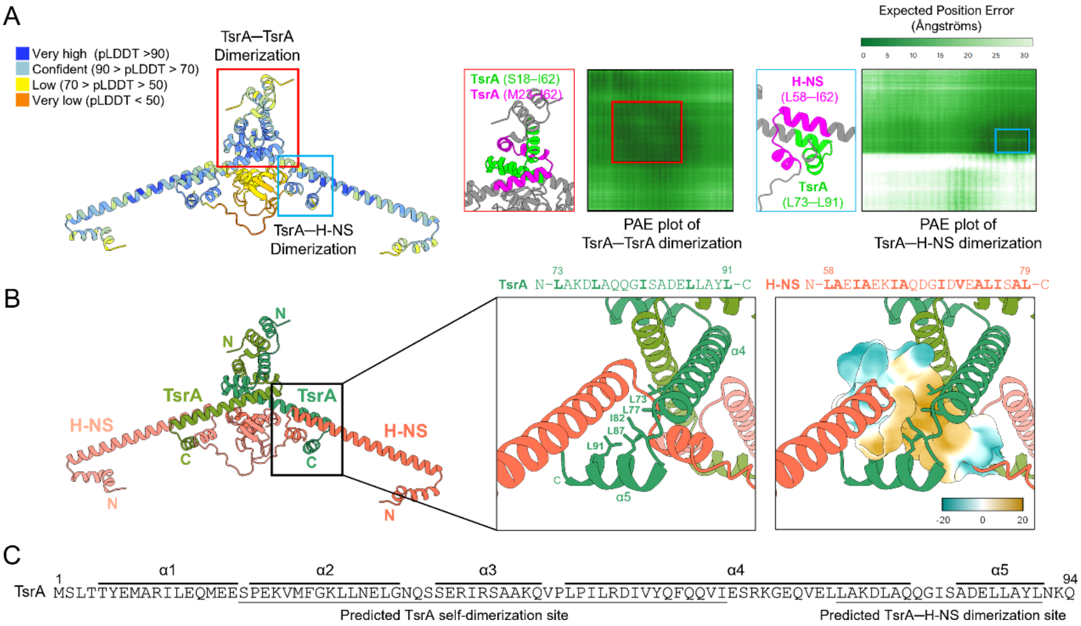

Fig S3. Structural modeling of the TsrA–H-NS complex predicted by AlphaFold3.

(A) Predicted heteromeric structure of H-NS/TsrA at 2:2 ratio, colored by pLDDT score with PAE plot showing a high confidence level (dark green) between TsrA–TsrA and TsrA–H-NS interfaces. The TsrA–TsrA dimerization has been suggested in *V. cholerae* (1, 2), aligned with our structural prediction. (B) The H-NS–TsrA complex with close-up view of the interaction interface. TsrA dimer acts as a bridge between two H-NS monomers. Residues of TsrA critical for its repressive activity, identified through the Ala/Ser mutational analysis in Fig 5, are indicated. These residues may interact with the hydrophobic surfaces of H-NS. Residues involved in hydrophobic interactions are in bold letters. The proteins were colored according to their polypeptide chains. (C) Amino acid sequence and predicted secondary structure of TsrA.

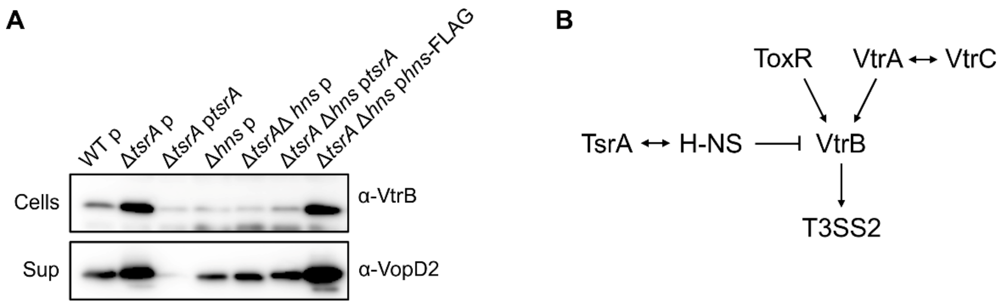

Fig S4. The regulatory activity of TsrA is dependent on H-NS.

(A) The indicated *V. parahaemolyticus* strains were grown in LB medium at 37°C to an OD_{600} of 1.8. Secreted proteins in culture supernatants (Sup) were analyzed by immunoblotting for VopD2, and bacterial cell lysates (Cells) were analyzed by immunoblotting for VtrB. Deletion of *hns* in the *tsrA* mutant (Δ*tsrA* Δ*hns*) abolished the increased expression of VtrB and enhanced T3SS2 secretion observed in Δ*tsrA*, resulting in a phenotype comparable to that of the *hns* mutant (Δ*hns*). This effect was restored by complementation with *hns*, but not with *tsrA*. (B) Model for regulation of T3SS2 gene expression. VtrA forms a complex with VtrC and, together with ToxR activates *vtrB* transcription, leading to VtrB-dependent induction of T3SS2 gene expression. In contrast, H-NS, acting in concert with TsrA, represses *vtrB* transcription.

REFERENCES

1. Caro F, Caro JA, Place NM, Mekalanos JJ. 2020. Transcriptional silencing by tsra in the evolution of pathogenic *Vibrio cholerae* biotypes. mBio 11: e02901-20.
2. Rakibova Y, Dunham DT, Seed KD, Freddolino L. 2024. Nucleoid-associated proteins shape the global protein occupancy and transcriptional landscape of a clinical isolate of *Vibrio cholerae*. mSphere 5: e01014-20.
