# Supplementary material for: TsrA modulates type III secretion system 2 expression as a co-regulator of H-NS in Vibrio parahaemolyticus
Source: J Bacteriol. 2026 May 28;208(6):e00556-25. doi: 10.1128/jb.00556-25 (PMC13277298; doi:10.1128/jb.00556-25)
Supplement: Tables S2 to S5 — Tables S2–S5. [file jb.00556-25-s0003.pdf]

Table S2. *V. parahaemolyticus* strains used in this study

**Strains**	**Genotypes/descriptions**	**References**
RIMD2210633 (WT)	Clinical isolate, serotype O3:K6, *tdh*$^{+}$ and T3SS2^{+}	Laboratory collection
Δ*tsrA*	RIMD2210633 *tsrA* (*vp3018*)	This study
Δ*vtrB*	RIMD2210633 *vtrB* (*vpa1348*)	(1)
Δ*vtrB* Δ*tsrA*	RIMD2210633 *vtrB tsrA*	This study
Δ*hns*	RIMD2210633 *hns* (*vp1133*)	(2)
Δ*hns* Δ*tsrA*	RIMD2210633 *hns tsrA*	This study
HNS-3F	RIMD2210633 *hns*::*hns-3xFLAG*	(2)
POR-2	RIMD2210633 *tdhA tdhS vcrD1* (T3SS1^{-})	(3)
POR-2 Δ*tsrA*	POR-2 *tsrA*	This study
ΔT3SS2	POR-2 *vcrD2*	(4)

Table S3. *E. coli* strains used in this study

**Strains**	**Genotypes/descriptions**	**References**
DH5α	F⁻ Φ80Δ*lacZ*M15 Δ(*lacZYA argF*)U169 *deoP recA1 endA1 hsdR17*(rK⁻ mK⁻)	Laboratory collection
BW19851	F⁻ *RP4-2*(*Km::Tn7,Tc::Mu-1*) Δ*uidA3::pir+ recA1 endA1 thiE1 hsdR17 creC510*	Laboratory collection
BL21(DE3)	F⁻ *omp*T, *hsdSB* (*r*B⁻, *m*B⁻), *dcm*, *gal*, λ(DE3)	Laboratory collection

Table S4. Plasmids used in this study

Plasmids	Description	References
pYAK1	oriR6K-based suicide vector for gene replacement, Cm^R	(5)
pYAK-Δ*tsrA*	A derivative of pYAK1 for deletion of *tsrA* gene	This study
pSA19CP	Complementation vector for *V. parahaemolyticus*, Cm^R	(6)
p*tsrA*	pSA19CP containing *tsrA* gene with its 300 bp upstream region	This study
p*tsrA*-His_6	p*tsrA* derivative carrying *tsrA* with a C-terminal 6×His tag	This study
p*tsrA*Δ*C*-His_6	p*tsrA*-His_6 derivative expressing C-terminally truncated *tsrA* (1–68)-6×His	This study
p*tsrA*-Q69A-His_6	p*tsrA*-His_6 with Q69A mutation	This study
p*tsrA*-V70A-His_6	p*tsrA*-His_6 with V70A mutation	This study
p*tsrA*-E71A-His_6	p*tsrA*-His_6 with E71A mutation	This study
p*tsrA*-L72A-His_6	p*tsrA*-His_6 with L72A mutation	This study
p*tsrA*-L73A-His_6	p*tsrA*-His_6 with L73A mutation	This study
p*tsrA*-A74S-His_6	p*tsrA*-His_6 with A74S mutation	This study
p*tsrA*-K75A-His_6	p*tsrA*-His_6 with K75A mutation	This study
p*tsrA*-D76A-His_6	p*tsrA*-His_6 with D76A mutation	This study
p*tsrA*-L77A-His_6	p*tsrA*-His_6 with L77A mutation	This study
p*tsrA*-A78S-His_6	p*tsrA*-His_6 with A78S mutation	This study
p*tsrA*-Q79A-His_6	p*tsrA*-His_6 with Q79A mutation	This study
p*tsrA*-Q80A-His_6	p*tsrA*-His_6 with Q80A mutation	This study
p*tsrA*-G81A-His_6	p*tsrA*-His_6 with G81A mutation	This study
p*tsrA*-I82A-His_6	p*tsrA*-His_6 with I82A mutation	This study
p*tsrA*-S83A-His_6	p*tsrA*-His_6 with S83A mutation	This study
p*tsrA*-A84S-His_6	p*tsrA*-His_6 with A84S mutation	This study
p*tsrA*-D85A-His_6	p*tsrA*-His_6 with D85A mutation	This study
p*tsrA*-E86A-His_6	p*tsrA*-His_6 with E86A mutation	This study
p*tsrA*-L87A-His_6	p*tsrA*-His_6 with L87A mutation	This study
p*tsrA*-L88A-His_6	p*tsrA*-His_6 with L88A mutation	This study
p*tsrA*-A89S-His_6	p*tsrA*-His_6 with A89S mutation	This study
p*tsrA*-Y90A-His_6	p*tsrA*-His_6 with Y90A mutation	This study

Plasmids	Description	References
p*tsrA*-L91A-His_6	p*tsrA*-His_6 with L91A mutation	This study
p*tsrA*-N92A-His_6	p*tsrA*-His_6 with N92A mutation	This study
p*tsrA*-K93A-His_6	p*tsrA*-His_6 with K93A mutation	This study
p*tsrA*-Q94A-His_6	p*tsrA*-His_6 with Q94A mutation	This study
p*hns*-FLAG	pSA19CP containing *hns* gene with its 500 bp upstream region and a C-terminal 3×FLAG tag	(2)
pET28a	Expression vector for *E. coli*, Km^R	Novagen
pET28a-*tsrA*-His_6	pET28a containing *tsrA* with a C-terminal 6×His tag, for expression TsrA-6×His	This study
pET28a-*tsrA*ΔC-His_6	pET28a-*tsrA*-His_6 derivative expressing C-terminally truncated *tsrA* (1–68)-6×His	This study
pE-SUMO-*hns*-FLAG	pE-SUMO-Kan containing *hns*-3×FLAG	This study

Table S5. Primers used in this study

**Primers**	**Sequence (5' to 3')**	**Description**
vtrB-promoter-F	CGCTGAGCCCTTTTCACAG	For Northern blotting and qRT-PCR
vtrB-promoter-R	GGAGTTTGAAGTGGTCGTC	
dVP3018/*tsrA*-1	GGGGATCCTTGGCACCGAAGTTCATGGAACG	For construction of pYAK-Δ*tsrA*
dVP3018/*tsrA*-2	GATTTCTTTTATTGCTTGTTTAGATAAGCCAGCAGAGCCATTTCATACGTGGTTAACGACATTAC	
dVP3018/*tsrA*-3	GTAATGTCGTTAACCACGTATGAAATGGCTCTGCTGGCTTATCTAAACAAGCAATAAAAGAAATC	
dVP3018/*tsrA*-4	GGCTGCAGCTGATTAGCCGCTACGACGAATACAC	
dc*tsrA*-F	TGGTTTGGCATTAACGCGGTCATC	For checking the deletion of *tsrA* locus in genome
dc*tsrA*-R	ACCGCGTAAGGAGGATACGATGACAG	
ups300-*tsrA*-F BamHI	GGGGATCCAACCATTCGGTGATAAAGCGGGCACAG	For construction of p*tsrA*
tsrA-R SalI	GGGTCGACTTATTGCTTGTTTAGATAAGCCAGCAGTTCATCCGC	
ups300-*tsrA*-F BamHI	GGGGATCCAACCATTCGGTGATAAAGCGGGCACAG	For construction of p*tsrA-His6*
tsrA-6xHis-R	GGGTCGACTTAATGGTGATGGTGATGGTGTTGCTTGTTTAGATAAGCCAGCAGTTCATCCGC	
ups300-*tsrA*-F BamHI	GGGGATCCAACCATTCGGTGATAAAGCGGGCACA	For construction of p*tsrA*Δ*C-His6*
tsrA-dC-6xHis-R SalI	GGGTCGACTTAATGGTGATGGTGATGGTGCTCGCCCTTGCGTGACTCAATCAC	
tsrA-F-NcoI	CCATGGATGTCGTTAACCACGTATGAAATGGCTCG	For construction of pET28a-*tsrA*-His6
tsrA-6xHis-R	GGGTCGACTTAATGGTGATGGTGATGGTGTTGCTTGTTTAGATAAGCCAGCAGTTCATCCGC	
hns-3F (pET28a)-F	GTTTAACTTTAAGAAGGAGATATACCATGTCAGAGCTGACTAAAACAC	For construction of p*hns*-FLAG
hns-3F (pET28a)-R	GCAAGCTTGTCGACCTACTTGTCATCGTCATCCTTG	

Primers	Sequence (5’ to 3’)	Description
pE-SUMO-*hns*-F	GGGGTCTCAAGGTATGTCAGAGCTGACTAAAA CACTTC	For purification of H-NS-FLAG (construction of pE-SUMO-*hns*-FLAG)
hns-3F (pET28a)-R	GCAAGCTTGTCGACCTACTTGTCA TCGTCATCCTTG	
pSA19-*tsrA* Q69A F	GAGTCACGCAAGGGCGAGGCAGTTGAGCTATT AGCAAAAGAC	For single amino acid mutation of *tsrA*, Q69A
pSA19-*tsrA* Q69A R	GTCTTTTGCTAATAGCTCAACTGCCTCGCCCTT GCGTGACTC	
pSA19-*tsrA* V70A F	TCACGCAAGGGCGAGCAAGCTGAGCTATT AGCA	For single amino acid mutation of *tsrA*, V70A
pSA19-*tsrA* V70A R	TGCTAATAGCTCAGCTTGCTCGCCCTTGCG TGA	
pSA19-*tsrA* E71A F	CGCAAGGGCGAGCAAGTTGCGCTATTAGCAA AAGACCTC	For single amino acid mutation of *tsrA*, E71A
pSA19-*tsrA* E71A R	GAGGTCTTTTGCTAATAGCGCAACTTGCTCGC CCTTGCG	
pSA19-*tsrA* L72A F	CGCAAGGGCGAGCAAGTTGAGGCATTAGCAA AAGACCTCGCA	For single amino acid mutation of *tsrA*, L72A
pSA19-*tsrA* L72A R	TGCGAGGTCTTTTGCTAATGCCTCAACTTGCTC GCCCTTGCG	
pSA19-*tsrA* L73A F	AAGGGCGAGCAAGTTGAGCTAGCAGCAAAAG ACCTCGCA	For single amino acid mutation of *tsrA*, L73A
pSA19-*tsrA* L73A R	TGCGAGGTCTTTTGCTGCTAGCTCAACTTGCTC GCCCTT	
pSA19-*tsrA* A74S F	GGCGAGCAAGTTGAGCTATTAAGCAAAGACCT CGCACAACAAGGT	For single amino acid mutation of *tsrA*, A74S
pSA19-*tsrA* A74S R	ACCTTGTTGTGCGAGGTCTTTGCTTAATAGCTC AACTTGCTCGCC	
pSA19-*tsrA* K75A F	GGCGAGCAAGTTGAGCTATTAGCAGCAGACCT CGCACAACAA	For single amino acid mutation of *tsrA*, K75A
pSA19-*tsrA* K75A R	TTGTTGTGCGAGGTCTGCTGCTAATAGCTCAA CTTGCTCGCC	
pSA19-*tsrA* D76A F	GGCGAGCAAGTTGAGCTATTAGCAAAAGCCCT CGCACAACAAGGT	For single amino acid mutation of *tsrA*, D76A
pSA19-*tsrA* D76A R	ACCTTGTTGTGCGAGGGCTTTTGCTAATAGCTC AACTTGCTCGCC	

Primers	Sequence (5' to 3')	Description
pSA19-*tsrA* L77A F	GAGCTATTAGCAAAAGACGCCGCACAACAAG GTATCTCAGCG	For single amino acid mutation of *tsrA*, L77A
pSA19-*tsrA* L77A R	CGCTGAGATACCTTGTTGTGCGGCGTCTTTTGC TAATAGCTC	
pSA19-*tsrA* A78S F	GCAAAAGACCTCAGCCAACAAGGTATCTCAGC GGATGAACTGCTG	For single amino acid mutation of *tsrA*, A78S
pSA19-*tsrA* A78S R	CAGCAGTTCATCCGCTGAGATACCTTGTTGGC TGAGGTCTTTTGC	
pSA19-*tsrA* Q79A F	GAGCTATTAGCAAAAGACCTCGCAGCACAAG GTATCTCAGCG	For single amino acid mutation of *tsrA*, Q79A
pSA19-tsrA Q79A R	CGCTGAGATACCTTGTGCTGCGAGGTCTTTTG CTAATAGCTC	
pSA19-*tsrA* Q80A F	GAGCTATTAGCAAAAGACCTCGCACAAGCAG GTATCTCAGCGGAT	For single amino acid mutation of *tsrA*, Q80A
pSA19-*tsrA* Q80A R	ATCCGCTGAGATACCTGCTTGTGCGAGGTCTT TTGCTAATAGCTC	
pSA19-*tsrA* G81A F	GCAAAAGACCTCGCACAACAAGCTATCTCAGC GGATGAA	For single amino acid mutation of *tsrA*, G81A
pSA19-*tsrA* G81A R	TTCATCCGCTGAGATAGCTTGTTGTGCGAGGT CTTTTGC	
pSA19-*tsrA* I82A F	GCAAAAGACCTCGCACAACAAGGTGCCTCAG CGGATGAACTG	For single amino acid mutation of *tsrA*, I82A
pSA19-*tsrA* I82A R	CAGTTCATCCGCTGAGGCACCTTGTTGTGCGA GGTCTTTTGC	
pSA19-*tsrA* S83A F	CTCGCACAACAAGGTATCGCAGCGGATGAACT GCTG	For single amino acid mutation of *tsrA*, S83A
pSA19-*tsrA* S83A R	CAGCAGTTCATCCGCTGCGATACCTTGTTGTG CGAG	
pSA19-*tsrA* A84S F	GACCTCGCACAACAAGGTATCTCAAGCGATGA ACTGCTGGCTTATC	For single amino acid mutation of *tsrA*, A84S
pSA19-*tsrA* A84S R	GATAAGCCAGCAGTTCATCGCTTGAGATACCT TGTTGTGCGAGGTC	
pSA19-*tsrA* D85A F	CAAGGTATCTCAGCGGCAGAACTGCTGGCTTA TCTA	For single amino acid mutation of *tsrA*, D85A
pSA19-*tsrA* D85A R	TAGATAAGCCAGCAGTTCTGCCGCTGAGATAC CTTG	

Primers	Sequence (5' to 3')	Description
pSA19-*tsrA* E86A F	GGTATCTCAGCGGATGCACTGCTGGCTTATCTAAAC	For single amino acid mutation of *tsrA*, E86A
pSA19-*tsrA* E86A R	GTTTAGATAAGCCAGCAGTGCATCCGCTGAGATACC	
pSA19-*tsrA* L87A F	CAAGGTATCTCAGCGGATGAAGCGCTGGCTTATCTAAACAAGCAA	For single amino acid mutation of *tsrA*, L87A
pSA19-*tsrA* L87A R	TTGCTTGTTTAGATAAGCCAGCGCTTCATCCGCTGAGATACCTTG	
pSA19-*tsrA* L88A F	GGTATCTCAGCGGATGAACTGGCGGCTTATCTAAACAAGCAA	For single amino acid mutation of *tsrA*, L88A
pSA19-*tsrA* L88A R	TTGCTTGTTTAGATAAGCCGCCAGTTCATCCGCTGAGATACC	
pSA19-*tsrA* A89S F	GGTATCTCAGCGGATGAACTGCTGAGTTATCTAAACAAGCAACAC	For single amino acid mutation of *tsrA*, A89S
pSA19-*tsrA* A89S R	GTGTTGCTTGTTTAGATAACTCAGCAGTTCATCCGCTGAGATACC	
pSA19-*tsrA* Y90A F	GCGGATGAACTGCTGGCTGCTCTAAACAAGCAACACCAT	For single amino acid mutation of *tsrA*, Y90A
pSA19-*tsrA* Y90A R	ATGGTGTTGCTTGTTTAGAGCAGCCAGCAGTTCATCCGC	
pSA19-*tsrA* L91A F	GCGGATGAACTGCTGGCTTATGCAAACAAGCAACACCATCAC	For single amino acid mutation of *tsrA*, L91A
pSA19-*tsrA* L91A R	GTGATGGTGTTGCTTGTTTGCATAAGCCAGCAGTTCATCCGC	
pSA19-*tsrA* N92A F	GAACTGCTGGCTTATCTAGCCAAGCAACACCATCACCATCAC	For single amino acid mutation of *tsrA*, N92A
pSA19-*tsrA* N92A R	GTGATGGTGATGGTGTTGCTTGGCTAGATAAGCCAGCAGTTC	
pSA19-*tsrA* K93A F	CTGCTGGCTTATCTAAACGCGCAACACCATCACCATCAC	For single amino acid mutation of *tsrA*, K93A
pSA19-*tsrA* K93A R	GTGATGGTGATGGTGTTGCGCGTTTAGATAAGCCAGCAG	
pSA19-*tsrA* Q94A F	CTGCTGGCTTATCTAAACAAGGCACACCATCACCATCACCAT	For single amino acid mutation of *tsrA*, Q94A
pSA19-*tsrA* Q94A R	ATGGTGATGGTGATGGTGTGCCTTGTTTAGATAAGCCAGCAG	

REFERENCES

1. Kodama T, Gotoh K, Hiyoshi H, Morita M, Izutsu K, Akeda Y, Park KS, Cantarelli VV., Dryselius R, Iida T, Honda T. 2010. Two regulators of *Vibrio parahaemolyticus* play important roles in enterotoxicity by controlling the expression of genes in the Vp-PAI region. PLoS One 5:e8678.
2. Pratama A, Ishii E, Kodama T, Iida T, Matsuda S. 2023. The Xenogeneic Silencer Histone-Like Nucleoid-Structuring Protein Mediates the Temperature and Salinity-Dependent Regulation of the Type III Secretion System 2 in *Vibrio parahaemolyticus*. J Bacteriol 205:e0026622.
3. Park KS, Ono T, Rokuda M, Jang MH, Okada K, Iida T, Honda T. 2004. Functional characterization of two type III secretion systems of *Vibrio parahaemolyticus*. Infect Immun 72:6659–6665.
4. Kodama T, Rokuda M, Park K, Cantarelli VV, Matsuda S, Iida T, Honda T. 2007. Identification and characterization of VopT , a novel ADP-ribosyltransferase effector protein secreted via the *Vibrio parahaemolyticus* type III secretion system 2. 9:2598–2609.
5. Kodama T, Akeda Y, Kono G, Takahashi A, Imura K, Iida T, Honda T. 2002. The EspB protein of enterohaemorrhagic *Escherichia coli* interacts directly with α-catenin. Cell Microbiol 4:213–222.
6. Nomura T, Hamashima H, Okamoto K. 2000. Carboxy terminal region of haemolysin of *Aeromonas sobria* triggers dimerization. Microb Pathog 28:25–36.
